# Effect of population density of lettuce intercropped with rocket on productivity and land-use efficiency

**DOI:** 10.1371/journal.pone.0194756

**Published:** 2018-04-26

**Authors:** Camila Seno Nascimento, Arthur Bernardes Cecílio Filho, Juan Waldir Mendoza-Cortez, Carolina Seno Nascimento, Francisco Bezerra Neto, Leilson Costa Grangeiro

**Affiliations:** 1 Universidade Estadual Paulista (UNESP), Department of Plant Production, Campus Jaboticabal, São Paulo, Brazil; 2 Universidad Nacional Agraria La Molina, Facultad de Agronomía, Departamento de Fitotecnia. Av. La Molina s/n, La Molina, Lima, PerúVEE; 3 Universidade Federal Rural do Semi-Árido (UFERSA), Department of Plant Sciences, Mossoró, Rio Grande do Norte, Brazil; Chinese University of Hong Kong, HONG KONG

## Abstract

The objective of this study was to evaluate the influence of the spacing of lettuce rows on the production of a lettuce-rocket intercropping system over two growing seasons (11 August to 25 September 2011 and 12 January to 24 February 2012) in Jaboticabal, São Paulo, Brazil. We evaluated 11 treatments in each season: lettuce-rocket intercrops with five row spacings for the lettuce (0.20, 0.25, 0.30, 0.35 and 0.40 m) and the rocket planted midway between the lettuce rows, sole crops of lettuce at the same five row spacings and a sole crop of rocket. Fresh and dry masses of the lettuce and rocket and number of lettuce leaves per plant were highest with a lettuce row spacing of 0.40 m, but the productivities of the lettuce and rocket were higher with a lettuce row spacing of 0.20 m. The productivities and fresh and dry weights of the lettuce and rocket and the number of lettuce leaves per plant were highest in the sole crops, but the fresh and dry weights of the rocket were higher with intercropping. The land equivalent ratios were >1.0 in both seasons in all intercrops and were highest for the densest crop (1.41). Intercropping was therefore 41% more efficient than sole cropping for the production of lettuce and rocket.

## Introduction

Modern agriculture has a considerable environmental impact, mainly due to intense soil management. Developing technologies that allow the rational use of land for food production is therefore necessary. Intercropping is an available technology that can assist in production but with less environmental impact. The production of vegetable crops on small and family farms, which are characterized by the intensive use of renewable and non-renewable resources, is an agricultural sector that can benefit from this agronomic practice due to the possibility of the cultivation of two or more species in the same area.

In addition to the environmental benefits of intercropping such as the reduction of cultivated area, the greater diversity of species per unit area and more extensive and faster growth of ground cover for decreasing soil erosion, intercropping may agronomically increase land-use efficiency and, consequently, food production per unit area, which could increase the profitability of agricultural activity [1–7]. Intercropping tends to have a higher productivity than traditional sole crops, mainly due to complementary resource use in time and space among different species [8]. But the efficiency of the intercropping system will depend on the choice of the crops and the management of the cultivation system for minimizing the competition between species for the environmental resources and for maximizing the complementarity between them [9].

Studies have indicated that the intercropping of lettuce (*Lactuca sativa*) and rocket (*Eruca sativa*) is feasible [10–14], but the spacing between cultures is an important factor because it affects both the spatial (when the shoots and roots of the crops capture the resources in different zones) and temporal (when the largest resource demands by the crops occur at different times) complementarity of the species and therefore the efficiency and viability of the combination. An increase in population density can thus increase the competition among species for light, water and nutrients and interfere upon the growth, productivity and quality of the vegetable crops.

The population densities of the species in intercropping have been rarely studied. We therefore evaluated the influence of the row spacing of lettuce on the productivity of lettuce intercropped with rocket over two growing seasons.

## Materials and methods

Two experiments were conducted from 11 August to 25 September 2011 and from 12 January to 24 February 2012, corresponding to the winter and summer seasons, respectively, in UNESP, *Campus* Jaboticabal, São Paulo, Brazil (21°15'22'' S, 48°18'58" W, 575 m a.s.l.). The mean maximum, mean minimum and average temperatures, relative humidity, rainfall and insolation during the experimental period were 31.3, 15.1 and 22.4°C, 52.6%, 11.9 mm and 265.3 h mo^-1^ in the winter and 30.4, 19.4 and 23.9°C, 77.2%, 189.6 mm and 207.3 h mo^-1^ in the summer, respectively.

The soil of the experimental area is a typical Rhodic Eutrudox [15]. A chemical analysis of the soil prior to the winter and summer seasons indicated a pH (CaCl_2_) of 5.5 and 5.2, 22 and 18 g dm^-3^ of organic matter, 96 and 99 mg dm^-3^ of P(resin), 3.8 and 4.4 mmol_c_ dm^-3^ of K, 33 and 33 mmol_c_ dm^-3^ of Ca, 9 and 10 mmol_c_ dm^-3^ of Mg, 18 and 34 mmol_c_ dm^-3^ of H^+^+Al, 63.8 and 81.4 mmol dm^-3^ cation exchange capacity and 72 and 58% base saturation of the soil, respectively.

Each experiment used a randomized complete block design with four replicates of 11 treatments. The treatments consisted of lettuce-rocket intercrops at five row spacings of 0.20, 0.25, 0.30, 0.35 and 0.40 m and the rocket planted midway between the lettuce rows, sole crops of lettuce at the same five spacings and a sole crop of rocket. The total area of the experimental unit was variable because of the different row spacings, but the beds always had nine rows and each row had four lettuce plants, totaling 36 plants per experimental unit. Data for the lettuce were collected from the ten most central plants, i.e. two plants from each of the five central rows. Data for the rocket were collected from the four central rows.

The soil was prepared by plowing and harrowing before the construction of the beds. Calcined lime (48% CaO and 16% MgO) was added 30 days before planting, with a relative power of total neutralization of 125%, to increase the base saturation of the soil to 80% [16].

Seedlings of the lettuce cultivar 'Vera', of the Crespa group, were transplanted at the four-leaf stage with 0.25 m between plants. The rocket cultivar 'Folha Larga' for the intercrop was directly sown on the day of lettuce transplantation in open furrows midway between the rows of lettuce. The rocket plants in the first and second experiments were thinned at 10 and 13 days after sowing (DAS), respectively, to adjust the spacing between plants to 0.05 m. The rocket sole crop had a row spacing of 0.20 m.

At planting, nutrient doses were applied for intercrop and sole crops of lettuce and rocket as recommended by Trani et al. [16] for lettuce and rocket monocultures. Also, side-dress applications of N (urea) followed the recommendation of Trani et al. [16] for sole crops the lettuce and rocket. The fertilizer was distributed along the rows, about 3 cm apart, 10, 20 and 30 days after transplantation of the lettuce and 7, 14 and 21 DAS for the rocket. The third N fertilization was not applied in both experiments to the intercrops with row spacings of 0.20, 0.25 and 0.30 m, because the crops completely covered the ground, preventing the application of the fertilizer.

Weeds were controlled during the experiments by manual hoeing. Phytosanitary treatments were also applied using products registered for the cultures. The crops were irrigated by sprinkler at a rate of 5 mm d^-1^. The lettuce was harvested when the plants in the sole crop at the row spacing of 0.25 m were ready for harvest, because this spacing is typical for lettuce in the Jaboticabal region. The rocket was harvested when the plants of the sole crop were >24 cm in height.

Total N content (g kg^-1^) for newly developed lettuce leaves (one per plant) was determined at the 2/3 point of the crop cycle, following Trani and Raij [17]. No recommendations are available for the assessment of nutritional status in rocket, so we determined N content in the shoot at harvest. Fresh mass (g plant^-1^) was determined immediately after harvest by weighing the plants on an electronic scale. The number of leaves per plant were obtained by count leaves (>5 cm in length) only for the lettuce. The dry mass of leaves (g plant^-1^) was obtained by oven-drying the leaves (both crops) with forced air at 65°C to a constant weight. Plant height (cm) was measured only for the rocket; 15 plants randomly selected prior to harvest were measured from the ground surface to the highest end of the foil, not extended. The lettuce was ranked into three classifications: first (<150 g plant^-1^), special (≥150 and <250 g plant^-1^) and extra (≥250 g plant^-1^). The lettuce plants were also evaluated for the presence of major and minor defects and were classified as extra, category I and category II. The rocket was not commercially classified, because no system was available in the literature, but we evaluated the presence of defects: deformed sheets, patches, decay and discoloration. The productivity of both cultures in kg m^-2^ was estimated based on a bed area of 6600 m^2^ ha^-1^.

The land equivalent ratio (LER) was calculated using the formula proposed by Willey [18]: *LER* = (*Yla*)/(*Yll*)+(*Yal*)/(*Yaa*) where *Yla* is the productivity of culture "l" intercropped with culture "a", *Yal* is the productivity of culture "a" intercropped with culture "l", *Yll* is the productivity of the lettuce sole crop and *Yaa* is the productivity of the rocket sole crop. A LER of 1 indicates no advantage of intercropping, a LER >1 indicates cooperation or compensation between intercrops, with advantages for the intercropping system, and a LER <1 indicates mutual inhibition or compensation, with disadvantages of intercropping relative to sole cropping.

Individual analyses of variance were conducted for both growing seasons, and a joint analysis was conducted using AgroEstat [19] when the ratio of the residual mean squares did not exceed seven times. The analysis of variance for the lettuce was conducted using the 2 x 5 factorial scheme, where the studied factors were both cropping systems (sole and intercropping) and the five spacings between lettuce rows. The analysis of variance for the rocket was conducted for the six treatments (five intercrops and one sole crop) containing rocket.

## Results and discussion

### Lettuce

Cropping system and spacing had no effect on N content, alone or in an interaction. Only the growing season had a significant effect. The average foliar N contents were 44.0 and 38.1 g kg^-1^ in winter and summer, respectively, both within the normal range for lettuce (30–50 g kg^-1^) reported by Trani and Raij [17]. N fertilization of the intercrops thus did not affect the N content of the lettuce sole crop. That is, the N fertilization for the rocket did not benefit the lettuce and the N fertilization for the lettuce did not benefit the rocket to the point of changing the N content of the lettuce. The fertilization was therefore not a differentiating factor between the cropping systems for promoting the growth of the lettuce.

The number of lettuce leaves (NL) was significantly affected by the interaction between the factors evaluated and the growing season. The lettuce plants had 22.5% more leaves in summer than in winter. The temperature regime and solar radiation in the summer were likely instrumental in promoting the growth of new lettuce leaves. Barbosa et al. [14] also reported a higher number of leaves for lettuce (15.4%) in the autumn than in winter for three types of lettuce intercropped with rocket.

Number of leaves in winter was not influenced by the interaction between cultivation system and row spacing but was influenced by cropping system. Number of leaves was higher for the sole crop (19.8) than the intercrop (17.0). Interactions between the factors, cropping systems and spacings had significant effects on number of leaves in the summer. Apart of the interaction, lettuce number of leaves was higher in the sole crops, and number of leaves decreased with row spacing within each cultivation system (Table 1 and S1 Table).

**Table 1:** Mean values of the number of leaves per plant, fresh mass, dry mass, productivity, and percentage of plants in the special and extra classes of lettuce as a function of row spacing and cropping system in winter and summer.

Cropping system	Row spacing (m)				
	0.20	0.25	0.30	0.35	0.40
	**Number of leaves per plant**				
	**Summer**				
**Intercropped**	16.75 bC*	19.00 bBC	21.25 bB	24.25 aA	26.25 aA
**Sole crop**	21.00 aC	22.75 aBC	23.50 aBC	24.50 aAB	26.75 aA
	**Fresh mass (g plant**^**-1**^**)**				
	**Summer**				
**Intercropped**	145.19 bD	177.27 bC	229.72 bB	314.93 bA	333.08 bA
**Sole crop**	249.13 aC	235.16 aC	333.07 aB	360.79 aA	368.43 aA
	**Dry mass (g plant**^**-1**^**)**				
	**Winter**				
**Intercropped**	16.10 bC	18.79 bCD	21.47 Bb	25.22 bB	30.22 bA
**Sole crop**	28.12 aBC	24.50 bC	32.82 aAB	31.60 aAB	33.77 aA
	**Summer**				
**Intercropped**	19.18 bB	20.41 bB	21.98 bB	22.53 bB	28.73 bA
**Sole crop**	25.47 aB	26.25 aB	28.40 aB	29.72 aB	34.57 aA
	**Productivity (kg m**^**-2**^**)**				
	**Winter**				
**Intercropped**	3.05 bA	2.74 bAB	2.25 bB	2.38 bAB	2.58 bAB
**Sole crop**	5.91 aA	4.60 aB	4.44 aB	3.94 aBC	3.38 aC
	**Summer**				
**Intercropped**	2.91 bC	2.84 bC	3.05 bBC	3.60 bA	3.33 bAB
**Sole crop**	4.98 aA	3.76 aC	4.43 aB	4.13 aB	3.68 aC
	**Special Class (%)**				
	**Summer**				
**Intercropped**	37.50 aB	90.63 aA	62.50 aAB	0.00 aC	3.13 aC
**Sole crop**	56.25 aA	65.63 bA	18.75 bB	6.25 aB	0.00 aB
	**Extra Class (%)**				
	**Winter**				
**Intercropped**	0.00 bB	0.00 bB	0.00 bB	15.63 bB	59.37 bA
**Sole crop**	84.37 aA	84.37 aA	87.50 aA	93.75 aA	93.75 aA
	**Summer**				
**Intercropping**	0.00 bC	0.00 bC	34.37 bB	100.00 aA	96.87 aA
**Sole crop**	43.75 aB	34.37 aB	81.25 aA	93.75 aA	100.00 aA

^*^ Different upper and lowercase letters within a row and column, respectively, indicate significant differences by a Tukey's test at 5% probability.

**Fig 1:**
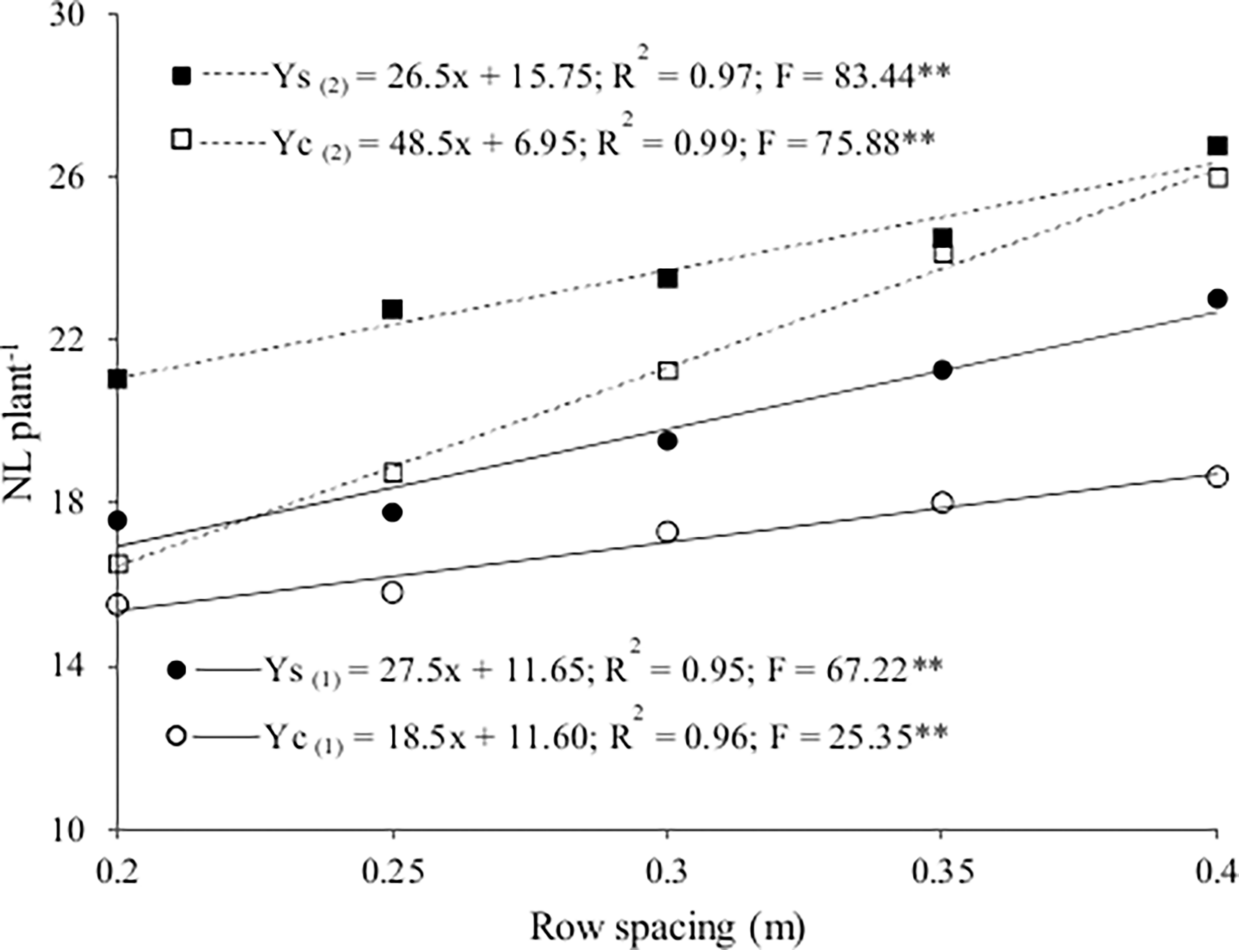
Number of leaves per plant (NL) as a function of lettuce row spacing for the sole crop (Ys) and the intercrop (Yc) in winter (1) and summer (2).

Number of leaves increased linearly with row spacing in both growing seasons (Fig 1 and S1 Table) and was higher in the sole crop at the lowest population density (0.4 m spacing), both in winter (22.65) and summer (26.35) relative to the intercrops. The presence of the rocket in the intercrops with lettuce affected the growth of the lettuce at the higher population densities (lower spacings between rows) in both seasons, indicated by lower number of leaves, likely because of increased competition for resources, especially for light.

Oliveira et al. [11] also reported that number of leaves for 'Vera' lettuce was higher in a sole crop (16.7) than when intercropped with rocket (mean of 14.5 from four spatial arrangements). In contrast, Barbosa et al. [14] found no differences in number of leaves for three types of lettuce (smooth, crisp and American) among sole crops and when intercropped with rocket (direct seeding), regardless of growing season.

The interaction of cropping system and row spacing with season were significant for the fresh mass of lettuce. Fresh mass was higher in summer (274.69 g plant^-1^) than in winter (257.68 g plant^-1^). The difference between the fresh masses was not large but was probably due to the greater thermal availability in the summer, which favored the best development of the lettuce plants. Fresh mass of lettuce was not influenced by the interaction between cropping system and row spacing in winter but was influenced by each factor separately. Fresh mass was 39.2% higher in the sole crop (320.44 g plant^-1^) than the intercrop. Fresh mass was significantly influenced by the interaction between cropping system and spacing in the summer. Fresh mass was higher in the sole crop than the intercrop for all spacings (Table 1 and S1 Table) and increased in both cropping systems as row spacing increased. Fresh mass of lettuce was highest at spacings of 0.35 and 0.40 m, which did not differ significantly.

**Fig 2:**
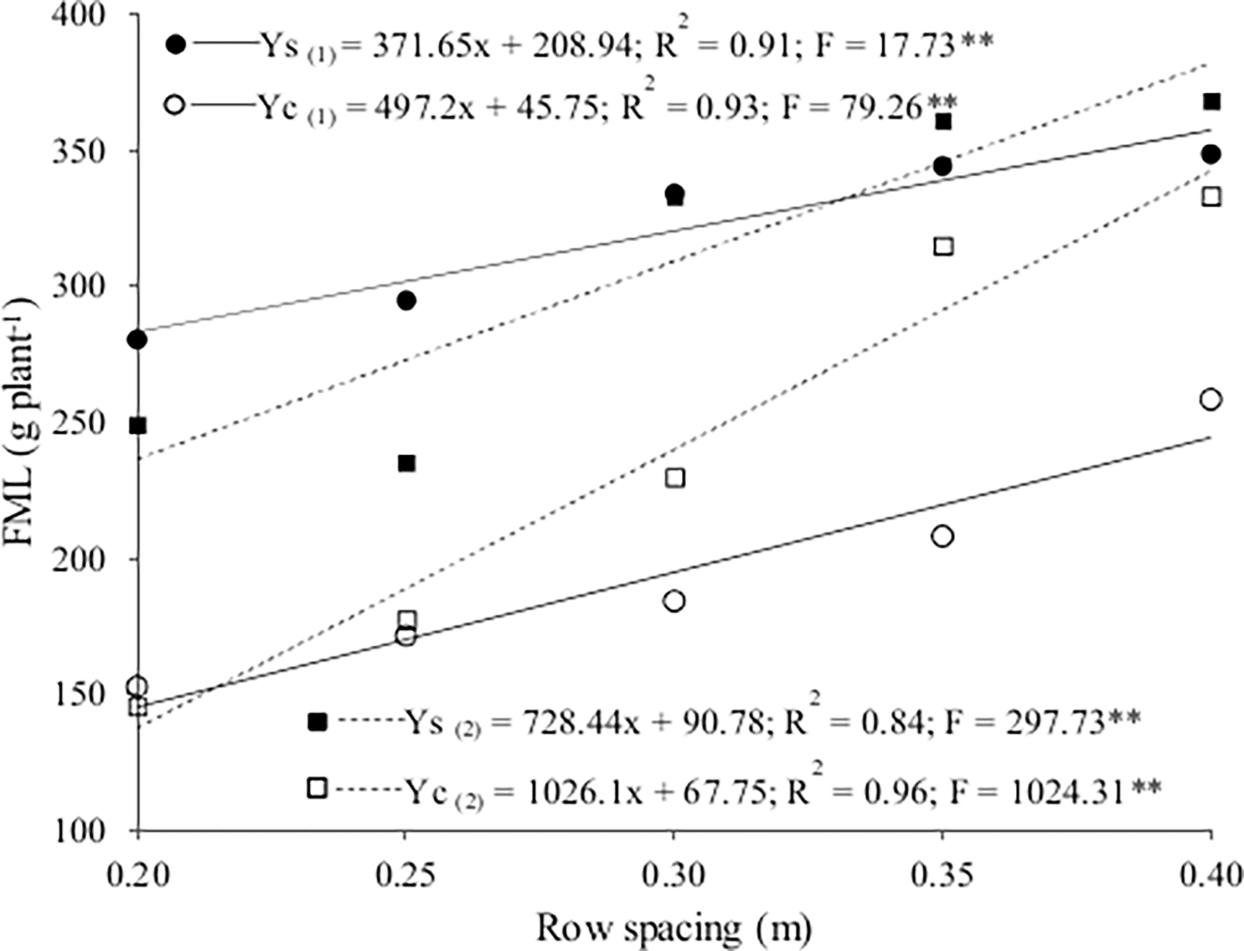
Fresh mass of lettuce (FML) as a function of lettuce row spacing for the sole crop (Ys) and the intercrop (Yc) in winter (1) and summer (2).

Fresh mass of lettuce increased linearly with row spacing in both seasons and cropping systems (Fig 2 and S1 Table). It was highest in the sole crop at the lowest population density, at 357.6 g plant^-1^ in winter and 382.2 g plant^-1^ in summer. Fresh mass of lettuce increased by 3.7 and 7.2 g plant^-1^ for the sole crop and by 4.9 and 10.3 g plant^-1^ for the intercrop in winter and summer, respectively, for each extra centimeter of spacing from 0.20 to 0.40 m. The rate of increase in fresh mass was highest for the intercrop in both seasons, likely due to increases in the intensity of inter- and intraspecific competition for environmental resources, especially at the higher population densities.

Barros Júnior et al. [12] also reported a higher fresh mass of lettuce in a sole crop than in an intercrop with rocket in the spring. Barbosa et al. [14] reported that fresh mass was higher with intercropping (crisp lettuce and rocket) than sole cropping in the autumn but not winter. Costa et al. [10] reported that fresh mass was higher in spring (501.1 g plant^-1^) than in autumn-winter (316.7 g plant^-1^) but did not differ significantly between sole cropping and intercropping with rocket.

**Fig 3:**
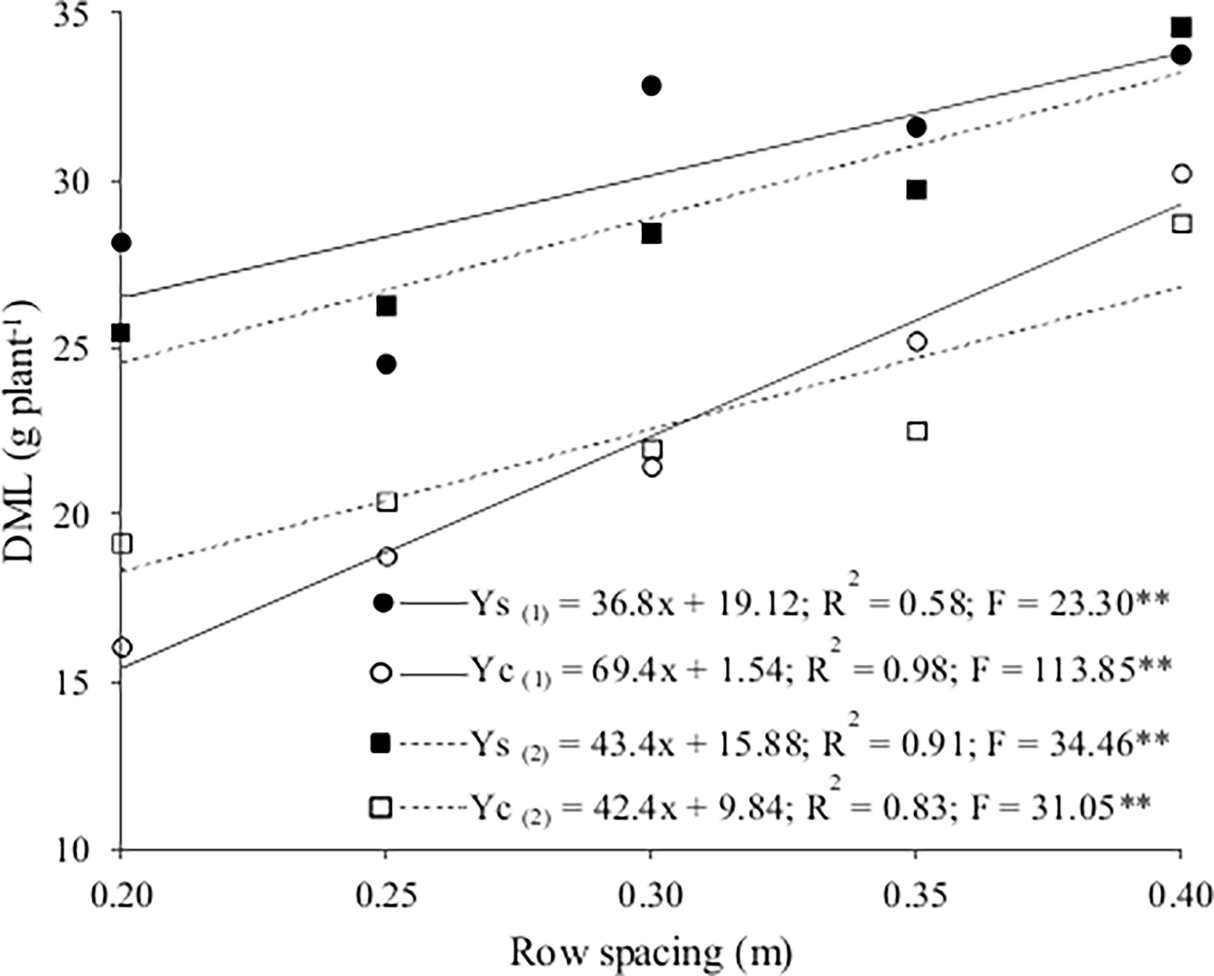
Dry mass of lettuce (DML) as a function of lettuce row spacing for the sole crop (Ys) and the intercrop (Yc) in winter (1) and summer (2).

The interaction of cropping system and spacing with season had a significant effect on the dry mass of lettuce. In contrast to number of leaves and fresh mass, dry mass of lettuce (26.26 g plant^-1^) was higher in the winter than the summer, but the difference was only 2%. The interaction between cropping system and spacing also affected the dry mass of lettuce in both season. It was higher in both seasons in the sole crop than the intercrop and increased with row spacing (Table 1 and S1 Table). The dry mass of lettuce increased linearly with row spacing in both seasons and cropping systems (Fig 3 and S1 Table). As noted for fresh mass, dry mass was highest at the spacing of 0.4 m in the sole crop at 33.8 g plant^-1^ in winter and 33.2 g plant^-1^ in summer. Costa et al. [10] reported a higher dry mass of lettuce in spring (30.2 g plant^-1^) than in autumn-winter (14.9 g plant^-1^), but the dry mass did not differ significantly between sole cropping and intercropping with rocket. Oliveira et al. [11] also found no differences in the dry mass of lettuce between sole cropping and intercropping with rocket.

The observed increases in the number of leaves, and in the fresh and dry mass of lettuce in intercropping as function of bigger spacing between rows can be attributed to the greater spatial complementarity between the lettuce and rocket species. In this case, this spatial complementarity is explained for the best use of incident solar radiation. As in the present study, the availability of nutrients was not limiting, attested by the leaf N content in lettuce, and water was available to the plants daily through irrigation, the main limiting factor for the growth of the plants was the intercepted solar intensity. This factor of production is mentioned by Sinoquet and Caldwell [20] like the main limiting factor for the establishment, growth and production by crops in this cropping system when the demands of the plant by nutrients and water are attended. With the increase in population density there is less space for growth of the plants, whose leaf area decreases in relation to another plant with greater spacing and, consequently, less solar interception per plant occurs. According to Monteith [21, 22], there is a direct relation between solar interception and the accumulation of fresh and dry mass.

Bigger growth of lettuces in sole crop than in intercropping is explained for the same reasons. While in sole crop has only intraspecific competition in intercropping has interspecific competition too. In intercropping, the ability of one species to capture resources will usually be constrained by competition for space with the other species [23].

**Fig 4:**
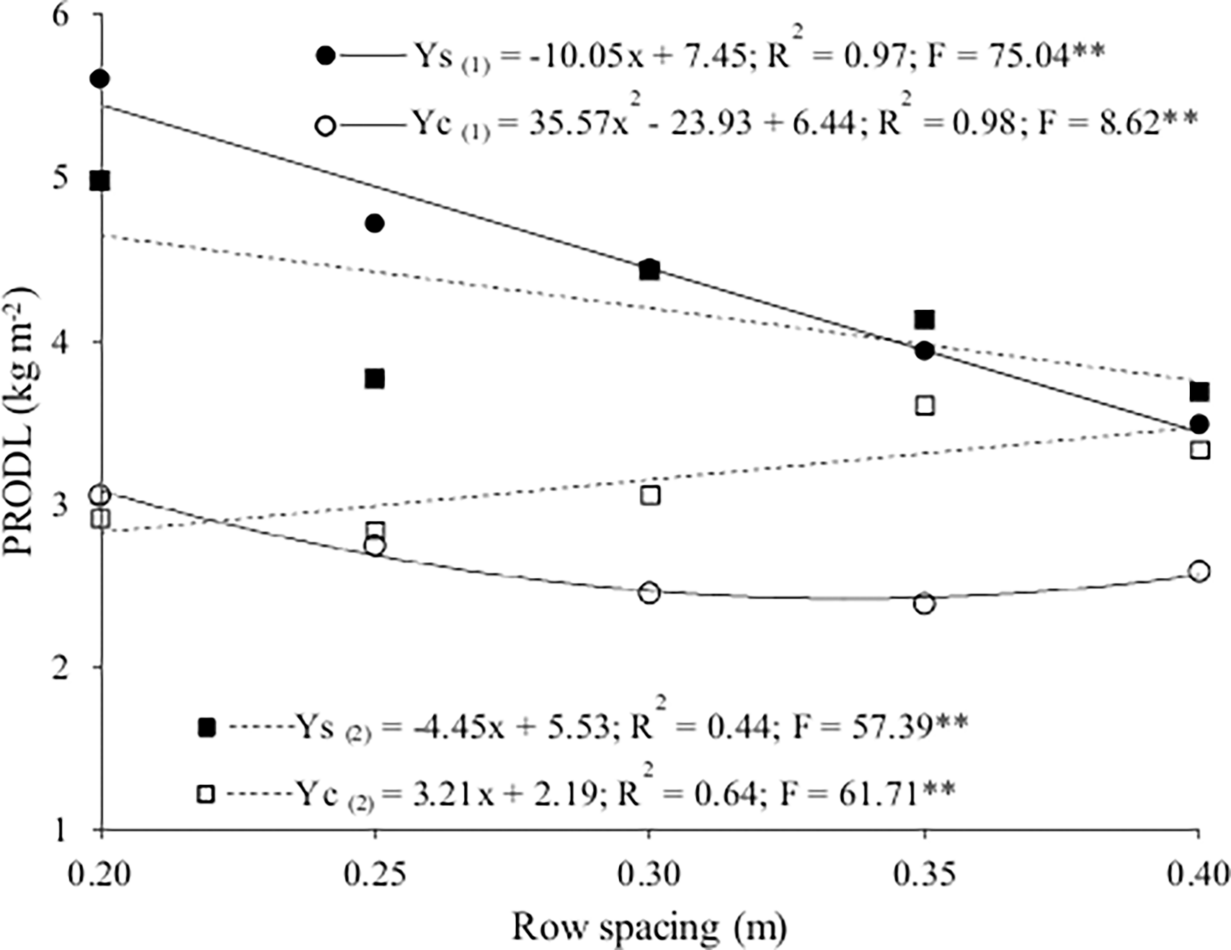
Productivity of lettuce (PRODL) as a function of lettuce row spacing for the sole crop (Ys) and the intercrop (Yc) in winter (1) and summer (2).

The productivity of lettuce was significantly affected by the interaction between cropping system and spacing with growing season. The productivity was higher in summer (3.67 kg m^-2^) than in winter (3.54 kg m^-2^). The productivity of lettuce was higher in both seasons in the sole crop than the intercrop and decreased as row spacing increased (Table 1 and S1 Table). Lower productivity of lettuce in intercropping is explained by intra- and interspecific competition between plants by resources of production while in sole crop only intraspecific competition. Bigger row spacing enable to plants improve their capture of resources and so increase spacial complementary [23]. Productivity decreased quadratically with row spacing in the winter intercrop but linearly in the sole crop. Productivity in summer decreased linearly with row spacing in the sole crop but increased linearly in the intercrop (Fig 4 and S1 Table). Productivity for the sole crop was 37 and 19% higher in winter (5.4 kg m^-2^) and summer (4.6 kg m^-2^), respectively, at the highest (0.20 m between rows) than at the lowest (0.40 m between rows) population density. Productivity was lower in both seasons for the intercrop than the sole crop.

The interactions between the treatment factors and season were not significant for the first class of the lettuce classification. The intercropping system at the smallest spacing (0.20 m), however, produced a higher percentage of first class lettuce, likely due to the higher intra- and interspecific competition, which decreased the weights of the lettuce. The sole cropping system at 0.25 m between rows produced the fewest first class plants, about 1.7% of the total produced, due to the absence of interspecific competition, which provided more room for growth.

The interactions between the treatment factors and season was significant for the special class. This class in winter was not influenced by the interaction between cropping system and spacing. The cultivation system nevertheless affected this trait. The sole crop produced fewer special class plants than the intercrop. The interaction between the cropping system and spacing, however, was significant in the summer. Intercropping performed better than sole cropping at spacings of 0.25, 0.30 and 0.40 m (Table 1 and S1 Table). The spacing of 0.25 m produced the most special class plants in both cropping systems.

The interaction between the treatment factors and season were significant for the extra class. Cropping system and spacing significantly affected the production of extra class plants in both seasons. The sole crop produced more extra class plants than the intercrop in the winter and summer, due to the absence of interspecific competition for resources, which provided more room for the growth of the lettuce. The intercropping system only produced lettuces in this category at row spacings ≥0.30 m in summer and ≥0.35 m in winter (Table 1 and S1 Table).

Intercropped lettuce, particularly at spacings between 0.20 and 0.30 m, had some bent and yellowing leaves, so belonged to category II of lesser value. Symptoms were mild or did not occur at larger spacings. Sole cropped lettuce, however, had no defects and was thus classified as extra. Intercropping with rocket thus decreased not only lettuce productivity but also quality, expressed as both lower class and category.

### Rocket

The rocket foliar N content was not influenced by the interaction between the treatments and season but differed between winter (51.0 g kg^-1^) and summer (46.3 g kg^-1^). The foliar N content therefore did not differ between sole cropped rocket (47.7 g kg^-1^) and rocket intercropped with lettuce (48.9 g kg^-1^), regardless of the lettuce row spacing and season. We assume that the fertilizer applied for the lettuce was not used by the rocket, and vice versa, as stated above.

The interactions between the treatments and seasons had no significant effect on the height of the rocket plants. The cultivation system alone also did not influence the height (Table 2 and S2 Table), as also observed by Oliveira et al. [11] at row spacings of 21.1 cm for an intercrop and 20.9 cm for a sole crop (20.9 cm). Season, however, had a significant effect on height, which was higher in summer (28.05 cm) than winter (25.95 cm).

**Table 2:** Mean values of the height, fresh mass (FMR), and dry mass (DMR) of rocket as a function of cropping system in winter and summer.

Cropping system	Height (cm)	FMR (g m^-1^)	DMR (g m^-1^)	
		Summer	Winter	Summer
**Intercrop with 0.20 m between rows**	27.82 a*	169.90 d	18.48 ab	12.90 c
**Intercrop with 0.25 m between rows**	26.88 a	245.05 c	18.83 ab	14.72 bc
**Intercrop with 0.30 m between rows**	26.70 a	233.73 c	20.00 a	13.16 c
**Intercrop with 0.35 m between rows**	27.58 a	292.63 b	20.28 a	18.87 a
**Intercrop with 0.40 m between rows**	26.02 a	384.70 a	14.30 b	18.59 ab
**Sole cropped rocket**	26.98 a	301.42 b	14.56 b	15.95 abc

* Means followed by the same letter within a column do not differ by a Tukey's test at 5% probability.

**Fig 5:**
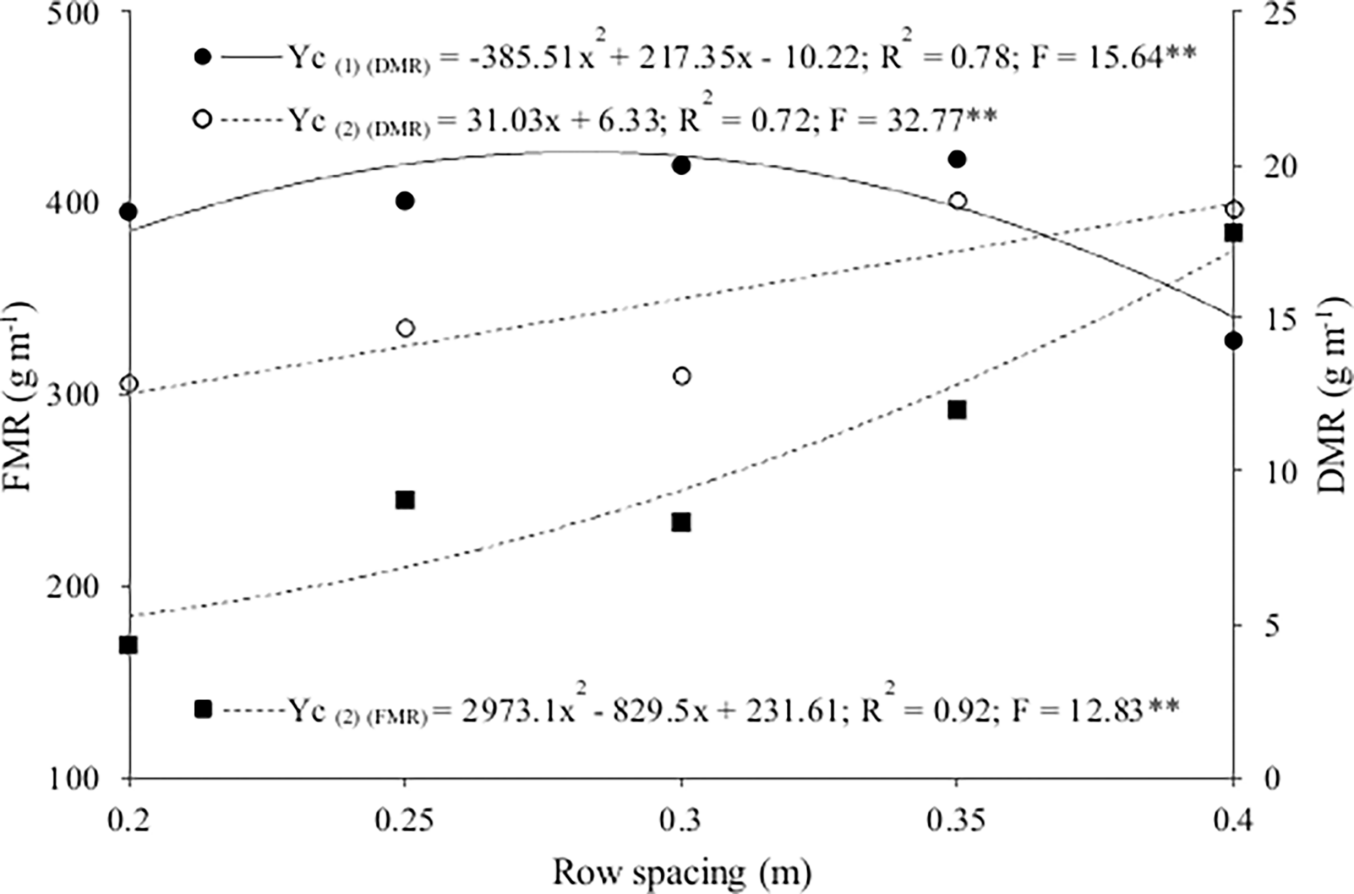
Fresh mass (FMR) and dry mass (DMR) of rocket intercropped (Yc) with lettuce as a function of lettuce row spacing in winter (1) and summer (2).

The interaction between the treatments and seasons had a significant effect on the fresh mass of the rocket. The cropping system had no influence on fresh mass in the winter, with an average of 259.82 g m^-1^, but significantly affected fresh mass in the summer. It was higher in the intercropping system at the row spacing of 0.40 m than in the sole crop at a spacing of 0.20 m (Table 2 and S2 Table). The rocket was sown midway between the lettuce rows, so at the intercropped row spacing of 0.40 m, the distance between the rocket and lettuce plants was 0.20 m, the same as the distance between rocket rows in the sole crop. The high fresh mass of rocket at the intercropped row spacing of 0.40 m may thus have been due to the slower growth of the lettuce relative to the rocket, allowing the rocket to grow with less competition for environmental resources. At the intercropping lettuce row spacing of 0.35 m, fresh mass of rocket did not differ significantly from that obtained in the sole crop and was lower than that when the intercrops were established with smaller spacings between the lettuce rows. Fresh mass increased quadratically with lettuce row spacing in the intercropping system in summer (Fig 5 and S2 Table). It was highest (375.5 g m^-1^) at the lowest population density (larger spacing), 51% higher than at the lowest density and 20% higher than for the sole crop (301.42 g m^-1^).

Results for seasonal fresh mass of rocket reported in the literature are inconsistent. Barbosa et al. [14] observed a higher fresh mass in a sole crop than in intercropping with different lettuce cultivars (smooth, crisp and American) in both the fall and winter. Costa et al. [10], however, found no differences in fresh mass between sole cropping (186.0 g m^-1^) and intercropping (253.7 g m^-1^) in an autumn-winter cultivation when the rocket was sown on the same day as crisp lettuce transplantation. These authors also found no differences in fresh mass between sole cropping (475.8 g m^-1^) and intercropping (440.5 g m^-1^) in the spring, regardless of the time of the sowing of the rocket relative to the transplantation of crinkly lettuce. Similarly, Barros Júnior et al. [12] found no difference in fresh mass between sole cropping and intercropping with crisp lettuce in the spring.

The interactions of the treatment factors and seasons in our study significantly affected the dry mass of the rocket. The cropping system influenced dry mass in both seasons. It was highest in both seasons with intercropping at the lettuce row spacing of 0.35 m (Table 2 and S2 Table). Costa et al. [10] found no difference in dry mass between sole cropping (27.3 g m^-1^) and intercropping (26.9 g m^-1^) in the spring when the rocket was sown on the same day as crisp lettuce transplantation. These authors reported a higher dry mass with sole cropping (33.8 g m^-1^) than intercropping (27.4 g m^-1^) with crisp lettuce in an autumn-winter cultivation, regardless of the time of establishment of the lettuce and rocket.

Dry mass of rocket in the intercropping system varied quadratically in winter and linearly in summer with lettuce row spacing (Fig 5 and S2 Table). In winter it was highest (20.41 g m^-1^) at the row spacing of 0.28 m and lowest at 0.40 m (15.04 g m^-1^). In contrast, dry mass in the summer was highest (18.59 g m^-1^) and lowest (15.04 g m^-1^) at the spacings of 0.40 and 0.20 m, respectively.

The productivity of the rocket was not influenced by the interaction between the treatments and seasons but was affected by the season and cropping system. In contrast to productivity of lettuce, the productivity of rocket was 18% higher in winter (3.76 kg m^-2^) than in summer. Productivity was highest (4.82 kg m^-2^) with sole cropping and was approximately 14, 27, 38, 44 and 52% higher than the intercrops at the lettuce row spacings of 0.20, 0.25, 0.30, 0.35 and 0.40 m, respectively, likely due to the absence of interspecific competition in the sole crop. Productivity of rocket in the intercropping system decreased as lettuce row spacing increased. It (4.14 kg m^-2^) was 44% higher at the highest lettuce population density (smallest spacing) than at the lowest density (larger spacing).

Costa et al. [10] reported that productivity of rocket was higher with sole cropping than intercropping in 83% of cases in two growing seasons with crisp, smooth and American lettuce cultivars. Barbosa et al. [14] also obtained higher productivities with sole cropping than intercropping in two growing seasons with the same three lettuce cultivars, which ranged from 3.16 to 3.74 kg m^-2^ in autumn and from 1.78 to 1.90 kg m^-2^ in winter. Barros Júnior et al. [12] reported that productivity of rocket in the spring was higher with sole cropping (1.6 kg m^-2^) by approximately 31%.

### Land equivalent ratio

**Fig 6:**
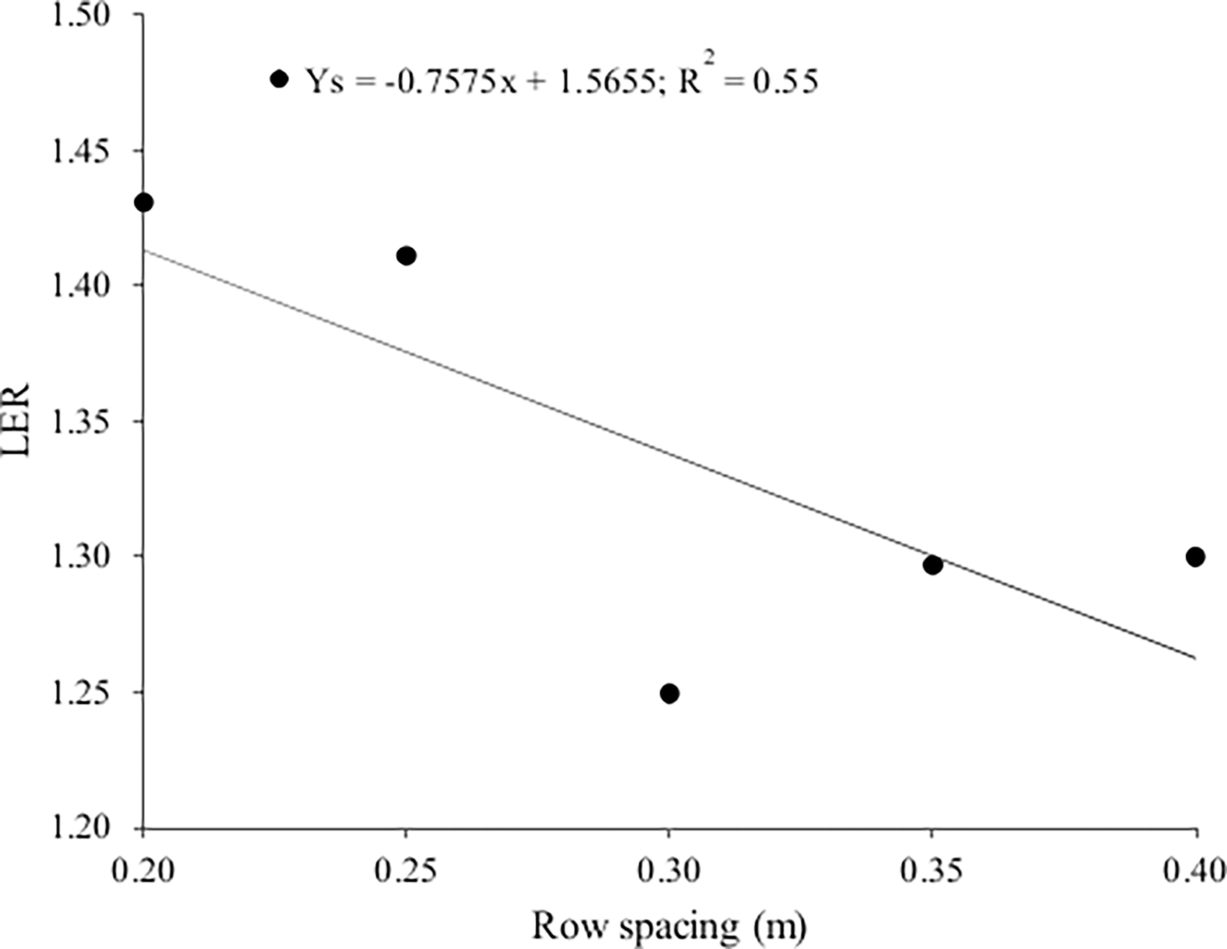
Land equivalent ratio (LER) as a function of lettuce row spacing.

The interaction between the treatments and seasons did not have a significant effect on the land equivalent ratio (LER), but season did. It was highest (1.44) in winter, 17.5% more efficient than in summer (1.23). Land equivalent ratio decreased linearly with increasing lettuce row spacing in both seasons (Fig 6 and S3 Table). It was high (1.41) at the smallest spacing (0.20 m); sole cropping would need 41% more land to produce the same amount as intercropping, i.e. 1 ha of intercropping would produce the equivalent of 1.41 ha of sole cropping. Thus intercropping made better use of the environmental resources and/or inputs than sole cropping, agreeing with Gou et al. [8], who says that intercropping tends to have a higher productivity than traditional sole crops, mainly due to complementary resource use in time and space among different species. Intercropping have greater ability to intercept solar radiation once they occupy the area better than sole crops [8]. According to Willey [24], the improved productivity can result from either greater interception of solar radiation, a higher light use efficiency, or a combination of the two. Light interception is sometimes increased as a result of growing two species together in one field [25] either as a result of a lengthening of the period of soil coverage (temporal advantage), or mainly in this case as a result of a more complete soil cover (spatial advantage).

Land equivalent ratio, however, decreased as row spacing increased, likely due mainly to the lower complementarity and higher competition between the species. In intercropping, the ability of one species to capture resources will usually be constrained by competition for space with the other species [26].

Costa et al. [10] reported ratios of 1.93 and 1.84 in the autumn-winter and spring, respectively, for three varieties of lettuce. Barros Júnior et al. [12] reported a higher land equivalent ratio (1.84) in the spring due to the effect of N fertilization on lettuce intercropped with rocket. Barbosa et al. [14] reported land equivalent ratios between 1.03 and 1.41 for three lettuce cultivars (smooth, crisp and American) intercropped with rocket in two growing seasons, which were higher in autumn than winter (between 0.72 and 1.0).

## Conclusions

Increasing the spacing between lettuce rows, in both the winter and summer, increased fresh and dry mass and leaf number of the lettuce and decreased the productivities of the lettuce and rocket. Fresh and dry mass, leaf number and the productivities of lettuce and rocket were lower with intercropping than sole cropping in both the winter and summer. Sole cropping produced a higher percentage of lettuce plants with better commercial classification in both the winter and summer. Fresh and dry mass of the intercropped rocket were highest at lettuce row spacings of 0.28 and 0.40 m in both the winter and summer. The land equivalent ratio was highest in the winter at the lettuce row spacing of 0.20 m.

## Supporting information

S1 Table Values observed of fresh mass, dry mass, number of leaves, productivity, first class, special class, extra class and foliar nitrogen of lettuce plants as function of planting time, cultivation system and spacing between lettuce rows. (DOCX)

S2 Table Values observed of fresh mass, dry mass, productivity, height and foliar nitrogen of rocket plants as function of planting time, cultivation system and spacing between lettuce rows. (DOCX)

S3 Table Values observed of land use efficiency. (DOCX)
