# Supplementary material for: Effect of population density of lettuce intercropped with rocket on productivity and land-use efficiency
Source: PLoS One. 2018 Apr 26;13(4):e0194756. doi: 10.1371/journal.pone.0194756 (PMC5919433; doi:10.1371/journal.pone.0194756)
Supplement: S1 Table — (DOCX) [file pone.0194756.s001.docx]

**S1 Table. Values observed of fresh mass, dry mass, number of leaves, productivity, first class, special class, extra class and foliar nitrogen of lettuce plants as function of planting time, cultivation system and spacing between lettuce rows.**

| **Planting time** | **Cultivation**  **system** | **Spacing** | **Replicate** | **Fresh mass** | **Dry mass** | **Number of leaves** | **Productivity** | **First**  **class** | **Special class** | **Extra**  **class** | **Foliar nitrogen** |
| --- | --- | --- | --- | --- | --- | --- | --- | --- | --- | --- | --- |
|  |  |  |  | g planta^-1^ | g planta^-1^ | leaves plant^-1^ | kg m^-2^ | % of total | % of total | % of total | g kg^-1^ |
| Winter | Intercropping | 0.20 | 1 | 150.11 | 13.99 | 14.00 | 3.00 | 62.50 | 37.50 | 0.00 | 45.29 |
| Winter | Intercropping | 0.20 | 2 | 157.89 | 17.97 | 17.00 | 3.16 | 50.00 | 50.00 | 0.00 | 37.52 |
| Winter | Intercropping | 0.20 | 3 | 141.08 | 15.36 | 15.00 | 2.82 | 62.50 | 37.50 | 0.00 | 43.89 |
| Winter | Intercropping | 0.20 | 4 | 161.03 | 17.00 | 16.00 | 3.22 | 37.50 | 62.50 | 0.00 | 41.86 |
| Winter | Intercropping | 0.25 | 1 | 189.60 | 17.10 | 15.20 | 3.03 | 0.00 | 100.00 | 0.00 | 51.10 |
| Winter | Intercropping | 0.25 | 2 | 148.95 | 19.70 | 16.00 | 2.38 | 62.50 | 37.50 | 0.00 | 44.31 |
| Winter | Intercropping | 0.25 | 3 | 181.59 | 20.30 | 17.00 | 2.91 | 12.50 | 87.50 | 0.00 | 44.10 |
| Winter | Intercropping | 0.25 | 4 | 165.20 | 18.00 | 15.00 | 2.64 | 12.50 | 87.50 | 0.00 | 39.83 |
| Winter | Intercropping | 0.30 | 1 | 193.82 | 20.16 | 19.00 | 2.58 | 75.00 | 25.00 | 0.00 | 47.32 |
| Winter | Intercropping | 0.30 | 2 | 177.43 | 21.46 | 17.00 | 2.36 | 0.00 | 100.00 | 0.00 | 39.34 |
| Winter | Intercropping | 0.30 | 3 | 194.90 | 25.30 | 15.60 | 2.59 | 12.50 | 12.50 | 0.00 | 40.11 |
| Winter | Intercropping | 0.30 | 4 | 170.42 | 18.94 | 17.50 | 2.27 | 12.50 | 12.50 | 0.00 | 41.65 |
| Winter | Intercropping | 0.35 | 1 | 212.57 | 26.45 | 18.00 | 2.43 | 12.50 | 62.50 | 25.00 | 48.65 |
| Winter | Intercropping | 0.35 | 2 | 207.89 | 25.25 | 17.00 | 2.38 | 12.50 | 62.50 | 25.00 | 38.85 |
| Winter | Intercropping | 0.35 | 3 | 211.57 | 22.62 | 18.00 | 2.42 | 0.00 | 87.50 | 12.50 | 45.64 |
| Winter | Intercropping | 0.35 | 4 | 200.53 | 26.52 | 19.00 | 2.29 | 0.00 | 100.00 | 0.00 | 42.28 |
| Winter | Intercropping | 0.40 | 1 | 266.87 | 30.23 | 19.50 | 2.67 | 0.00 | 25.00 | 75.00 | 41.86 |
| Winter | Intercropping | 0.40 | 2 | 297.39 | 28.99 | 17.50 | 2.97 | 0.00 | 12.50 | 87.50 | 49.84 |
| Winter | Intercropping | 0.40 | 3 | 234.11 | 29.45 | 18.50 | 2.34 | 0.00 | 62.50 | 37.50 | 40.67 |
| Winter | Intercropping | 0.40 | 4 | 235.33 | 32.23 | 19.00 | 2.35 | 0.00 | 62.50 | 37.50 | 43.68 |
| Winter | Sole crop | 0.20 | 1 | 265.63 | 28.13 | 16.50 | 5.31 | 0.00 | 25.00 | 75.00 | 51.17 |
| Winter | Sole crop | 0.20 | 2 | 282.83 | 26.60 | 19.00 | 5.66 | 0.00 | 12.50 | 87.50 | 45.85 |
| Winter | Sole crop | 0.20 | 3 | 267.76 | 26.21 | 17.70 | 5.36 | 0.00 | 25.00 | 75.00 | 43.12 |
| Winter | Sole crop | 0.20 | 4 | 305.10 | 31.60 | 17.00 | 6.10 | 0.00 | 0.00 | 100.00 | 39.90 |
| Winter | Sole crop | 0.25 | 1 | 304.29 | 25.39 | 18.50 | 4.87 | 0.00 | 0.00 | 100.00 | 39.90 |
| Winter | Sole crop | 0.25 | 2 | 296.98 | 23.77 | 17.50 | 4.75 | 12.50 | 0.00 | 87.50 | 38.99 |
| Winter | Sole crop | 0.25 | 3 | 279.68 | 26.24 | 18.00 | 4.47 | 0.00 | 25.00 | 75.00 | 42.84 |
| Winter | Sole crop | 0.25 | 4 | 298.51 | 22.63 | 17.00 | 4.78 | 0.00 | 25.00 | 75.00 | 46.90 |
| Winter | Sole crop | 0.30 | 1 | 300.11 | 32.83 | 19.00 | 3.99 | 0.00 | 25.00 | 75.00 | 49.49 |
| Winter | Sole crop | 0.30 | 2 | 332.18 | 36.32 | 20.00 | 4.42 | 0.00 | 25.00 | 75.00 | 49.00 |
| Winter | Sole crop | 0.30 | 3 | 381.50 | 31.07 | 20.00 | 5.07 | 0.00 | 0.00 | 100.00 | 47.88 |
| Winter | Sole crop | 0.30 | 4 | 322.91 | 31.11 | 19.00 | 4.29 | 0.00 | 0.00 | 100.00 | 42.28 |
| Winter | Sole crop | 0.35 | 1 | 361.90 | 32.17 | 20.00 | 4.14 | 0.00 | 0.00 | 100.00 | 46.20 |
| Winter | Sole crop | 0.35 | 2 | 383.36 | 28.89 | 22.00 | 4.39 | 0.00 | 0.00 | 100.00 | 46.27 |
| Winter | Sole crop | 0.35 | 3 | 300.95 | 32.69 | 23.00 | 3.44 | 0.00 | 25.00 | 75.00 | 39.62 |
| Winter | Sole crop | 0.35 | 4 | 330.98 | 32.57 | 20.00 | 3.79 | 0.00 | 0.00 | 100.00 | 43.54 |
| Winter | Sole crop | 0.40 | 1 | 331.16 | 33.57 | 20.00 | 3.31 | 0.00 | 12.50 | 87.50 | 49.35 |
| Winter | Sole crop | 0.40 | 2 | 335.90 | 33.98 | 25.00 | 3.36 | 0.00 | 12.50 | 87.50 | 43.75 |
| Winter | Sole crop | 0.40 | 3 | 372.88 | 36.67 | 24.00 | 3.73 | 0.00 | 0.00 | 100.00 | 47.32 |
| Winter | Sole crop | 0.40 | 4 | 354.16 | 30.78 | 23.00 | 3.54 | 0.00 | 0.00 | 100.00 | 40.88 |
| Summer | Intercropping | 0.20 | 1 | 140.83 | 19.91 | 18.50 | 2.82 | 75.00 | 25.00 | 0.00 | 42.77 |
| Summer | Intercropping | 0.20 | 2 | 139.99 | 20.86 | 15.50 | 2.80 | 75.00 | 25.00 | 0.00 | 37.66 |
| Summer | Intercropping | 0.20 | 3 | 157.94 | 18.26 | 15.00 | 3.16 | 37.50 | 62.50 | 0.00 | 33.11 |
| Summer | Intercropping | 0.20 | 4 | 142.78 | 17.69 | 17.00 | 2.86 | 62.50 | 37.50 | 0.00 | 36.68 |
| Summer | Intercropping | 0.25 | 1 | 180.98 | 22.90 | 20.00 | 2.90 | 12.50 | 87.50 | 0.00 | 42.00 |
| Summer | Intercropping | 0.25 | 2 | 181.65 | 20.79 | 19.00 | 2.91 | 0.00 | 100.00 | 0.00 | 36.89 |
| Summer | Intercropping | 0.25 | 3 | 174.15 | 19.10 | 17.50 | 2.79 | 25.00 | 75.00 | 0.00 | 39.97 |
| Summer | Intercropping | 0.25 | 4 | 172.58 | 18.86 | 18.50 | 2.76 | 0.00 | 100.00 | 0.00 | 36.33 |
| Summer | Intercropping | 0.30 | 1 | 226.33 | 18.83 | 19.00 | 3.01 | 0.00 | 62.50 | 37.50 | 37.24 |
| Summer | Intercropping | 0.30 | 2 | 222.66 | 25.43 | 21.00 | 2.96 | 12.50 | 50.00 | 37.50 | 38.08 |
| Summer | Intercropping | 0.30 | 3 | 245.82 | 19.78 | 21.00 | 3.27 | 0.00 | 62.50 | 37.50 | 41.79 |
| Summer | Intercropping | 0.30 | 4 | 223.74 | 23.90 | 24.00 | 2.98 | 0.00 | 75.00 | 25.00 | 36.82 |
| Summer | Intercropping | 0.35 | 1 | 304.95 | 19.96 | 26.50 | 3.49 | 0.00 | 0.00 | 100.00 | 35.00 |
| Summer | Intercropping | 0.35 | 2 | 313.36 | 22.99 | 25.00 | 3.58 | 0.00 | 0.00 | 100.00 | 36.89 |
| Summer | Intercropping | 0.35 | 3 | 326.49 | 24.43 | 22.00 | 3.74 | 0.00 | 0.00 | 100.00 | 35.56 |
| Summer | Intercropping | 0.35 | 4 | 314.91 | 22.73 | 23.00 | 3.60 | 0.00 | 0.00 | 100.00 | 33.67 |
| Summer | Intercropping | 0.40 | 1 | 318.03 | 25.68 | 24.50 | 3.18 | 0.00 | 0.00 | 100.00 | 42.07 |
| Summer | Intercropping | 0.40 | 2 | 334.69 | 27.21 | 24.50 | 3.35 | 0.00 | 12.50 | 87.50 | 42.14 |
| Summer | Intercropping | 0.40 | 3 | 325.50 | 31.31 | 26.00 | 3.25 | 0.00 | 0.00 | 100.00 | 41.86 |
| Summer | Intercropping | 0.40 | 4 | 354.08 | 30.73 | 29.00 | 3.54 | 0.00 | 0.00 | 100.00 | 35.98 |
| Summer | Sole crop | 0.20 | 1 | 240.70 | 25.07 | 20.05 | 4.81 | 0.00 | 62.50 | 37.50 | 42.07 |
| Summer | Sole crop | 0.20 | 2 | 251.22 | 24.63 | 21.00 | 5.02 | 0.00 | 50.00 | 50.00 | 39.62 |
| Summer | Sole crop | 0.20 | 3 | 255.04 | 26.41 | 22.05 | 5.10 | 0.00 | 50.00 | 50.00 | 38.78 |
| Summer | Sole crop | 0.20 | 4 | 249.54 | 25.77 | 21.05 | 4.99 | 0.00 | 62.50 | 37.50 | 35.21 |
| Summer | Sole crop | 0.25 | 1 | 222.44 | 24.61 | 22.00 | 3.56 | 0.00 | 75.00 | 25.00 | 38.64 |
| Summer | Sole crop | 0.25 | 2 | 234.02 | 25.94 | 24.00 | 3.74 | 0.00 | 87.50 | 12.50 | 36.82 |
| Summer | Sole crop | 0.25 | 3 | 263.79 | 27.54 | 22.00 | 4.22 | 0.00 | 25.00 | 75.00 | 36.89 |
| Summer | Sole crop | 0.25 | 4 | 220.41 | 26.90 | 23.00 | 3.53 | 0.00 | 75.00 | 25.00 | 37.17 |
| Summer | Sole crop | 0.30 | 1 | 329.09 | 27.66 | 24.00 | 4.38 | 0.00 | 62.50 | 37.50 | 38.78 |
| Summer | Sole crop | 0.30 | 2 | 343.97 | 28.06 | 24.00 | 4.57 | 0.00 | 0.00 | 100.00 | 41.86 |
| Summer | Sole crop | 0.30 | 3 | 341.02 | 29.81 | 23.00 | 4.54 | 0.00 | 0.00 | 100.00 | 42.07 |
| Summer | Sole crop | 0.30 | 4 | 318.18 | 28.08 | 23.00 | 4.23 | 0.00 | 12.50 | 87.50 | 36.26 |
| Summer | Sole crop | 0.35 | 1 | 368.02 | 27.13 | 26.00 | 4.21 | 0.00 | 0.00 | 100.00 | 32.27 |
| Summer | Sole crop | 0.35 | 2 | 363.59 | 30.89 | 25.00 | 4.16 | 0.00 | 12.50 | 87.50 | 38.29 |
| Summer | Sole crop | 0.35 | 3 | 342.66 | 33.14 | 23.00 | 3.92 | 0.00 | 12.50 | 87.50 | 39.62 |
| Summer | Sole crop | 0.35 | 4 | 368.87 | 27.73 | 24.00 | 4.22 | 0.00 | 0.00 | 100.00 | 36.05 |
| Summer | Sole crop | 0.40 | 1 | 379.73 | 39.28 | 26.00 | 3.80 | 0.00 | 0.00 | 100.00 | 36.75 |
| Summer | Sole crop | 0.40 | 2 | 375.23 | 35.43 | 28.00 | 3.75 | 0.00 | 0.00 | 100.00 | 40.18 |
| Summer | Sole crop | 0.40 | 3 | 366.24 | 31.51 | 27.00 | 3.66 | 0.00 | 0.00 | 100.00 | 38.57 |
| Summer | Sole crop | 0.40 | 4 | 352.30 | 32.06 | 26.00 | 3.53 | 0.00 | 0.00 | 100.00 | 36.19 |
