# Supplementary material for: Effect of population density of lettuce intercropped with rocket on productivity and land-use efficiency
Source: PLoS One. 2018 Apr 26;13(4):e0194756. doi: 10.1371/journal.pone.0194756 (PMC5919433; doi:10.1371/journal.pone.0194756)
Supplement: S2 Table — (DOCX) [file pone.0194756.s002.docx]

**S2 Table. Values observed of fresh mass, dry mass, productivity, height and foliar nitrogen of rocket plants as function of planting time, cultivation system and spacing between lettuce rows.**

| **Planting time** | **Cultivation system** | **Spacing** | **Replicate** | **Fresh mass** | **Dry Mass** | **Productivity** | **Height** | **Foliar nitrogen** |
| --- | --- | --- | --- | --- | --- | --- | --- | --- |
|  |  |  |  | g planta^-1^ | g planta^-1^ | kg m^-2^ | cm | g kg^-1^ |
| Winter | Intercropping | 0.20 | 1 | 286.63 | 19.77 | 4.41 | 30.17 | 47.9 |
| Winter | Intercropping | 0.20 | 2 | 241.80 | 16.88 | 4.53 | 28.08 | 54.7 |
| Winter | Intercropping | 0.20 | 3 | 310.53 | 18.78 | 4.00 | 27.75 | 44.9 |
| Winter | Intercropping | 0.20 | 4 | 279.65 | 18.48 | 4.77 | 25.67 | 54.2 |
| Winter | Intercropping | 0.25 | 1 | 261.95 | 17.85 | 3.13 | 21.83 | 50.8 |
| Winter | Intercropping | 0.25 | 2 | 212.00 | 18.83 | 4.45 | 25.75 | 53.5 |
| Winter | Intercropping | 0.25 | 3 | 301.38 | 19.37 | 3.65 | 25.50 | 49.4 |
| Winter | Intercropping | 0.25 | 4 | 272.46 | 19.27 | 3.47 | 27.50 | 51.3 |
| Winter | Intercropping | 0.30 | 1 | 376.71 | 17.83 | 3.19 | 26.75 | 50.2 |
| Winter | Intercropping | 0.30 | 2 | 210.30 | 18.68 | 2.96 | 23.17 | 55.2 |
| Winter | Intercropping | 0.30 | 3 | 366.20 | 24.43 | 3.29 | 29.67 | 50.3 |
| Winter | Intercropping | 0.30 | 4 | 206.13 | 19.08 | 3.32 | 25.00 | 54.4 |
| Winter | Intercropping | 0.35 | 1 | 286.00 | 20.21 | 3.12 | 30.17 | 53.1 |
| Winter | Intercropping | 0.35 | 2 | 272.00 | 19.83 | 2.80 | 26.33 | 50.1 |
| Winter | Intercropping | 0.35 | 3 | 232.82 | 17.38 | 3.60 | 25.83 | 55.7 |
| Winter | Intercropping | 0.35 | 4 | 355.50 | 23.41 | 2.97 | 26.58 | 52.8 |
| Winter | Intercropping | 0.40 | 1 | 202.32 | 12.49 | 2.36 | 21.92 | 50.9 |
| Winter | Intercropping | 0.40 | 2 | 241.01 | 12.51 | 2.67 | 22.08 | 42.8 |
| Winter | Intercropping | 0.40 | 3 | 240.10 | 14.81 | 2.56 | 23.00 | 54.5 |
| Winter | Intercropping | 0.40 | 4 | 280.60 | 17.39 | 3.08 | 26.58 | 53.3 |
| Winter | Sole crop | 0.20 | 1 | 210.05 | 16.79 | 5.04 | 28.33 | 48.0 |
| Winter | Sole crop | 0.20 | 2 | 208.70 | 12.15 | 5.71 | 26.50 | 49.1 |
| Winter | Sole crop | 0.20 | 3 | 180.90 | 15.73 | 5.16 | 25.42 | 52.3 |
| Winter | Sole crop | 0.20 | 4 | 199.88 | 13.56 | 5.89 | 23.17 | 45.8 |
| Summer | Intercropping | 0.20 | 1 | 185.05 | 10.00 | 3.42 | 28.42 | 39.3 |
| Summer | Intercropping | 0.20 | 2 | 165.07 | 13.63 | 3.75 | 27.83 | 46.8 |
| Summer | Intercropping | 0.20 | 3 | 169.90 | 14.69 | 3.91 | 28.50 | 47.3 |
| Summer | Intercropping | 0.20 | 4 | 159.57 | 13.27 | 4.32 | 26.17 | 45.0 |
| Summer | Intercropping | 0.25 | 1 | 217.71 | 11.77 | 3.14 | 28.58 | 37.6 |
| Summer | Intercropping | 0.25 | 2 | 240.84 | 12.85 | 3.40 | 28.00 | 47.1 |
| Summer | Intercropping | 0.25 | 3 | 260.50 | 17.12 | 4.27 | 29.42 | 46.2 |
| Summer | Intercropping | 0.25 | 4 | 261.17 | 17.14 | 2.79 | 28.50 | 47.3 |
| Summer | Intercropping | 0.30 | 1 | 219.34 | 13.72 | 2.65 | 26.25 | 48.0 |
| Summer | Intercropping | 0.30 | 2 | 251.98 | 14.07 | 3.31 | 28.17 | 47.6 |
| Summer | Intercropping | 0.30 | 3 | 238.52 | 11.83 | 2.40 | 26.17 | 46.3 |
| Summer | Intercropping | 0.30 | 4 | 225.06 | 13.02 | 2.89 | 28.42 | 47.2 |
| Summer | Intercropping | 0.35 | 1 | 280.90 | 16.89 | 2.27 | 27.92 | 47.2 |
| Summer | Intercropping | 0.35 | 2 | 296.54 | 20.19 | 2.18 | 26.58 | 46.5 |
| Summer | Intercropping | 0.35 | 3 | 313.84 | 19.92 | 2.57 | 28.42 | 50.6 |
| Summer | Intercropping | 0.35 | 4 | 279.23 | 18.47 | 2.06 | 28.83 | 47.5 |
| Summer | Intercropping | 0.40 | 1 | 352.33 | 14.48 | 1.73 | 27.83 | 47.5 |
| Summer | Intercropping | 0.40 | 2 | 382.68 | 19.84 | 2.17 | 29.75 | 47.2 |
| Summer | Intercropping | 0.40 | 3 | 408.29 | 19.02 | 1.95 | 28.83 | 47.2 |
| Summer | Intercropping | 0.40 | 4 | 395.49 | 21.00 | 1.92 | 28.17 | 46.3 |
| Summer | Sole crop | 0.20 | 1 | 283.63 | 14.73 | 4.28 | 28.42 | 46.7 |
| Summer | Sole crop | 0.20 | 2 | 317.70 | 14.31 | 4.18 | 27.75 | 48.0 |
| Summer | Sole crop | 0.20 | 3 | 296.00 | 16.11 | 4.09 | 28.08 | 48.4 |
| Summer | Sole crop | 0.20 | 4 | 308.35 | 18.63 | 4.18 | 28.17 | 43.0 |
