# Supplementary material for: Effect of population density of lettuce intercropped with rocket on productivity and land-use efficiency
Source: PLoS One. 2018 Apr 26;13(4):e0194756. doi: 10.1371/journal.pone.0194756 (PMC5919433; doi:10.1371/journal.pone.0194756)
Supplement: S3 Table — (DOCX) [file pone.0194756.s003.docx]

**S3 Table. Values observed of land use efficiency.**

| **Planting time** | **Spacing** | **Replicate** | **Land Use Efficency** |
| --- | --- | --- | --- |
| Winter | 0.20 | 1 | 1.35 |
| Winter | 0.20 | 2 | 1.40 |
| Winter | 0.20 | 3 | 1.24 |
| Winter | 0.20 | 4 | 1.45 |
| Winter | 0.25 | 1 | 1.22 |
| Winter | 0.25 | 2 | 1.32 |
| Winter | 0.25 | 3 | 1.29 |
| Winter | 0.25 | 4 | 1.20 |
| Winter | 0.30 | 1 | 1.16 |
| Winter | 0.30 | 2 | 1.07 |
| Winter | 0.30 | 3 | 1.19 |
| Winter | 0.30 | 4 | 1.12 |
| Winter | 0.35 | 1 | 1.19 |
| Winter | 0.35 | 2 | 1.12 |
| Winter | 0.35 | 3 | 1.28 |
| Winter | 0.35 | 4 | 1.13 |
| Winter | 0.40 | 1 | 1.20 |
| Winter | 0.40 | 2 | 1.34 |
| Winter | 0.40 | 3 | 1.14 |
| Winter | 0.40 | 4 | 1.24 |
| Summer | 0.20 | 1 | 1.38 |
| Summer | 0.20 | 2 | 1.46 |
| Summer | 0.20 | 3 | 1.57 |
| Summer | 0.20 | 4 | 1.61 |
| Summer | 0.25 | 1 | 1.52 |
| Summer | 0.25 | 2 | 1.59 |
| Summer | 0.25 | 3 | 1.76 |
| Summer | 0.25 | 4 | 1.40 |
| Summer | 0.30 | 1 | 1.31 |
| Summer | 0.30 | 2 | 1.46 |
| Summer | 0.30 | 3 | 1.31 |
| Summer | 0.30 | 4 | 1.36 |
| Summer | 0.35 | 1 | 1.39 |
| Summer | 0.35 | 2 | 1.39 |
| Summer | 0.35 | 3 | 1.52 |
| Summer | 0.35 | 4 | 1.37 |
| Summer | 0.40 | 1 | 1.28 |
| Summer | 0.40 | 2 | 1.43 |
| Summer | 0.40 | 3 | 1.35 |
| Summer | 0.40 | 4 | 1.42 |
